# The study of ethnoveterinary medicinal plants at Mojana Wodera district, central Ethiopia

**DOI:** 10.1371/journal.pone.0267447

**Published:** 2022-05-25

**Authors:** Mikiyas Abebe

**Affiliations:** Faculty of Natural and computational sciences, Department of Biology, Woldia University, Woldia, Amhara region, Ethiopia; Mirpur University of Science and Technology, PAKISTAN

## Abstract

Ethnoveterinary study was conducted from March 2020 to December 2020 in Mojana Wodera district, centeral Ethiopia. The study was aimed to identify and document ethnoveterinary medicinal plant species and traditional medicinal knowledge of the traditional health practitioners. A total of 105 informants were selected purposely and volunteer sampling approaches, and from these total informants, 15 key informants were identified based on their knowledge difference. Semi-structured interviews, field observation, and discussion were employed to collect information. Descriptive statistical method was employed to analyze the collected data. Informant Consensus Factor (ICF) values were calculated to determine the most common livestock ailment categories that occurred and identify potentially effective medicinal plant species in respective disease categories. T-test was applied to compare knowledge difference. The result showed that a higher average (p< 0.05) was recorded for key informants, illiterate and elder group; however, there is no significance difference (p = 0.53) between gender. A total of 33 ethnoveterinary medicinal plant species, which belong to 23 families and 31 genera were identified. Family Asteraceae and family Solanaceae were the dominant. From this total number of plant species (12.12%) were endemic for Ethiopia. The finding showed that shrubs accounted for 39.39% followed by herbs (36.36%) and trees (15.15%). The medicinal plant parts that were most commonly utilized were leaf (55.36%) followed by root (23.21%) and seed (8.93%) respectively. Higher ICF was recorded for Blackleg (0.82) followed by general illness (0.8) and pasturalosis. In this study, *Vernonia amygdalina* was the most effective medicinal plants to treat blackleg.

## Introduction

As the economy of Ethiopia mainly depends on agriculture, the livestock sector contributes about 19% of the national GDP and 45% of agricultural GDP. At the same time, 16–19% of the foreign exchange earnings of the country depend on agriculture [1]. Similarly, for this core activity, the livestock sector has a great role [2]. According to [3,4], Ethiopia is one of the top ten countries in Africa in the wealth of livestock. It is estimated to be about 52 million cattle, 24.2 million sheep, 22.6 million goats, 5.7 million donkeys, 2 million horses, 1.1 million camels, and 45 million chickens [5]. Even though the country has plenty of livestock, which could be important for the economy of the country, the sector is not well developed. The main reason for this low development of the sector is different diseases that happened in the country [6].

Humans have used plants for centuries, and till now, numerous cultures have been used medicinal plants for their primary health care needs [7]. In Ethiopia, traditional remedies are the source of therapeutics for nearly 90% of livestocks. Regarding their origin, 95% are prepared from plants [8]. Like other developing countries, Ethiopian people prefer traditional medicinal plants to their livestock than the modern medical system. Inadequate veterinary health professionals, poor infrastructure, the cost of modern medicine, and the distance of veterinary health center from their home to rely on ethnoveterinary medicine than modern medical system [6,9].

As mentioned by [10–13], the indigenous knowledge of the people and medicinal plants are at risk by different factors such as deforestation, climate change, agricultural expansion, over-exploitation, habitat loss. Traditional medicinal knowledge of the people on medicinal plants is very crucial to treat different diseases. Even if some Ethiopian ethnoveterinary medicinal inventories have attempted to document veterinary medicinal plants in some cultural groups, it is not enough in comparison from the total of 85 different ethnolinguistic communities of the country. Similarly, in this study district, little attempt has been made to document ethnoveterinary medicinal plants by ethnomedicinal inventories. However till now, no research has been conducted regarding medicinal plants used to treat livestock in this district. Thus, this study aimed to fill this gap and to identify and document ethnoveterinary medicinal plant species and traditional medicinal knowledge of the traditional health practitioners in the study district.

## Materials and methods

### Description of the study area

The study was conducted in Mojana Wodera district, North Showa zone Amhara region, central Ethiopia. The district is far 202 km from the capital city of Ethiopia, Addis Ababa, and 72 km from administer center of North Showa, Debere Brehan town, in northern direction. It is bordered with Menze Mama Mider in the north, Menze Lalo Mider and Menze Keya Geberieal in the North West, Basona Worena in south and west, and Tarmaber district in the east. The elevation of the study district ranges between altitudes of 1459–3172 m above sea level, and traditionally, it is divided into three agricultural zones; Dega (28%), Woyna Dega (69%), and kola (3%). The annual rainfall of the district ranges from 800–1000 mm and the annual temperature ranges from 10−18^0^ C. The main administer center of the district is located at Seladengaye town that has historical significance as it has been the seat of Ethiopian emperor Menilik II.

### Demographic

The district has a total population of 83814 (44,489 (53.08% men) and 39325 (46.92% women), of whom 7799 (9.31%) urban inhabitant. The economic income of most people’s depends on both crop production and livestock. About 99.94% of the people are followers of Ethiopian orthodox Tewahedo Christianity (99.94%) and the remaining 0.06% is followers of Protestantism.

### Livestock population and veterinary service

The study District possesses about 345,170 livestock population; consisting of 58,250 (16.88%) cattle; 23424 (6.78%) equine; 160,202 (46.41%) sheep and goats and 103,294 (29.93%) poultry. In this study District, a total of thirteen veterinary clinics are available. However, still now most of the people are used traditional medicinal plants to treat their livestock from various ailments.

### Informant selection

A total of 105 informants (83 male and 22 female) were selected from seven district sites. Porposive and volunteer sampling approaches were applied to choose the representative general informants suggested by [14]. Fifteen key informants (11 male and 4 female) were selected based on suggestions from the members of the community, elder people and the informants themselves about their better knowledge. The age of the informants’ is ranged from 20–85 years (37 were between 20–39 years old, and the other 68 informants were above 40 years old).

### Data collection

Ethnoveterinary data was collected from March 19, 2020, to December 23, 2020 based on methods suggested by [15,16]. Accordingly, semi-structured interviews, field observation, discussion with informants, guided filed with informants, and walk-in-the-word were employed with their local language, Amharic, to collect information. Information regarding local names of medicinal plants, part of the plant used for medicine, methods of gathering, and preparation, the dosage used, diseases treated, route of application, use of the plant other than medicinal uses, and management methods were recorded at the spot. The collected plant species was dried, deep frozen, and identified at national herbarium of Ethiopia in Addis Ababa University.

### Data analysis

The collected ethno medicinal data were entered to excel spreadsheet software (Microsoft Corporation 2010) and organized for statistical analysis. The descriptive statistical method was employed to analyze and summarize the ethnobotanical data obtained from the interviews and observation such as medicinal value, methods of preparation, application, disease treated, route of application, the dosage of medicine, growth forms of plants, and parts of the plants. Traditional medicinal dynamics on use of medicinal plants by men and women, young and elder, illiterate and educated, key and general informants was compared using t- test (SPSS software, version 20) at 95% confidence level. Informant Consensus Factor (ICF) values were applied to determine the most common livestock ailment categories that occurred in the District and identify potentially effective medicinal plant species in respective disease categories as mentioned by [17,18]. The ICF was calculated as follows: Number of use citations in each category (n_ur_) minus the number of species used (nt), and divided by the numbers of use citations in each category minus one.


$$ICF=n_{ur}-nt/n_{ur}-1$$

Preference ranking was computed to evaluate the degree of effectiveness of certain medicinal plants against the most prevalent diseases in the study district.

## Results and discussion

### Results

#### Knowledge of the people on medicinal plants

Even though the average number of medicinal plants reported by male informants (2.53 ± 1.426) was higher than female respondents (2.00 ± 1.234), statistically the difference was non-significant (p > 0.05). The senior members of the community (40–85 years old) were reported significantly (p < 0.05) higher medicinal plants than youngest group (20–39 years old. Similarly, there was significant difference (p < 0.05) between illiterate and educated groups of the community in medicinal plants reported. Key informants knew significantly (p <0.05) higher number of medicinal plants than general informants (Table 1).

**Table 1:** Statistical test of knowledge among different groups of informants on average number of medicinal plants reported.

Parameters used	Groups of informants	N	Average ± SD	P value
Gender	Male	83	2.53 ± 1.426	0.53
	Female	22	2.00 ± 1.234	
Age	Youngest group (20–39 yrs old)	37	1.54 ± 0.767	0.000
	Senior group (40–85 yrs old)	68	2.90 ± 1.437	
Educational level	Illiterate	41	2.85 ± 1.131	0.01
	Educated	64	2.14 ± 1.489	
Informant category	Key informants	15	3.40 ± 1.502	0.003
	General informants	90	2.26 ± 1.320	

N = Number of respondents; significance difference (p <0.05).

The reference used in this table was the same as Table 2 of doi: 10.1186/1746-4269-10-21.

#### Gaining and transfer of indigenous medicinal plant knowledge

From the total of 105 practitioners, 76 (72.38%) acquired their medicinal plant knowledge from their family especially from their father’s and grandfather’s through oral tales with a high level of secrecy while the remaining 17 (16.19%) and 12 (11.43%) acquired their knowledge through trial and error, and from their friends as well as by reading different material respectively.

#### Diversity of ethnoveterinary medicinal plants in the district

**Fig 1:**
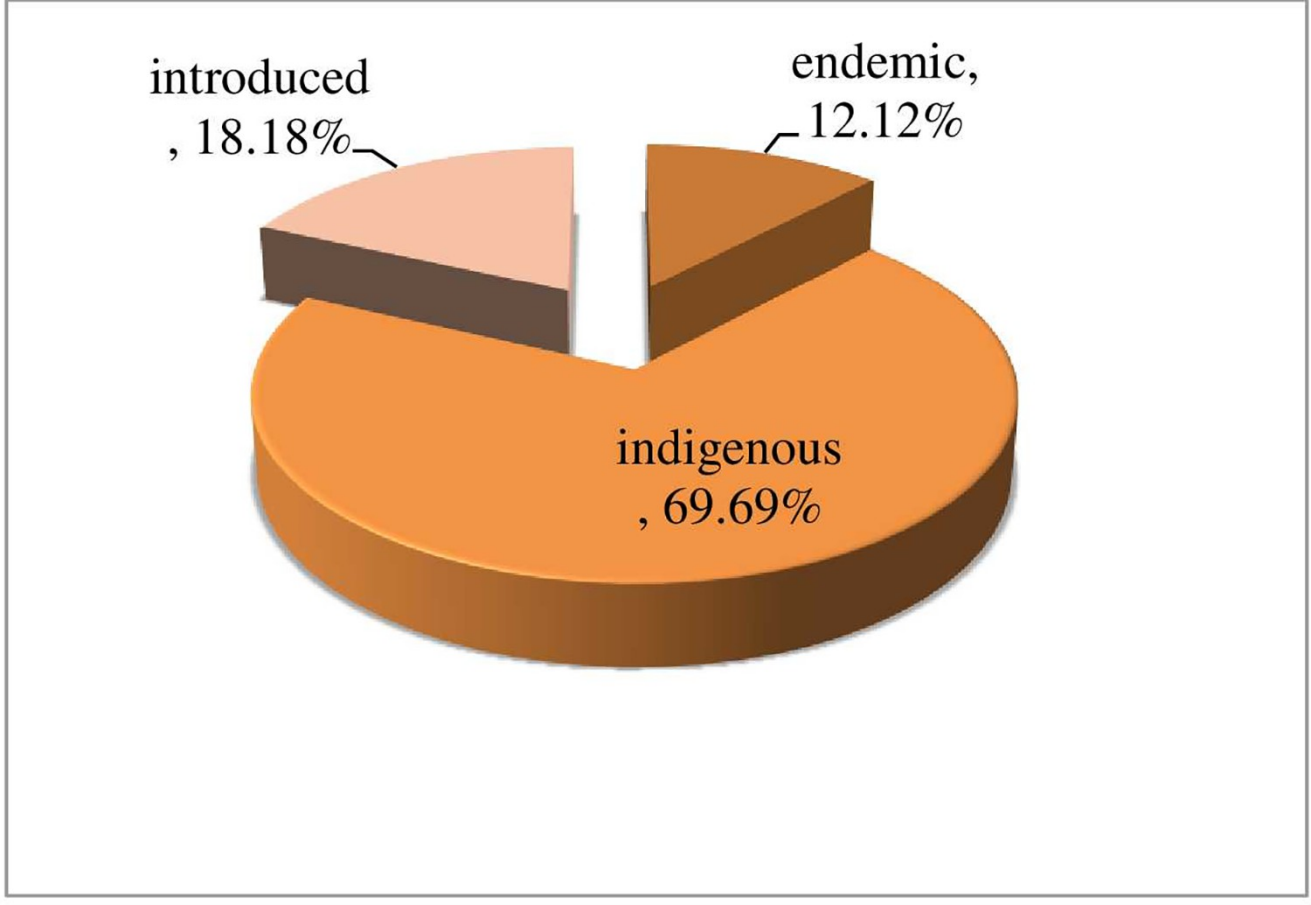
Plant endemism in the district.

A total of 33 ethnoveterinary medicinal plant species, which belong to 23 families, and 31 genera, were identified in the study district. Family Asteraceae and family Solanaceae were represented by 4 (12.12%) species each; followed by family Aloaceae, Cucurbitaceae, Fabaceae and Myrtaceae had 2 (6.06%) species each. The remaining 17 (51.52%) families were represented by a single species. From the total 33 plant species, 4 (12.12%) species are endemic, 23 (69.70%) species are indigenous and the remaining 6 (18.18%) species are introduced for Ethiopia (Fig 1) (S1 Table).

#### Growth habit and their habitat

**Fig 2:**
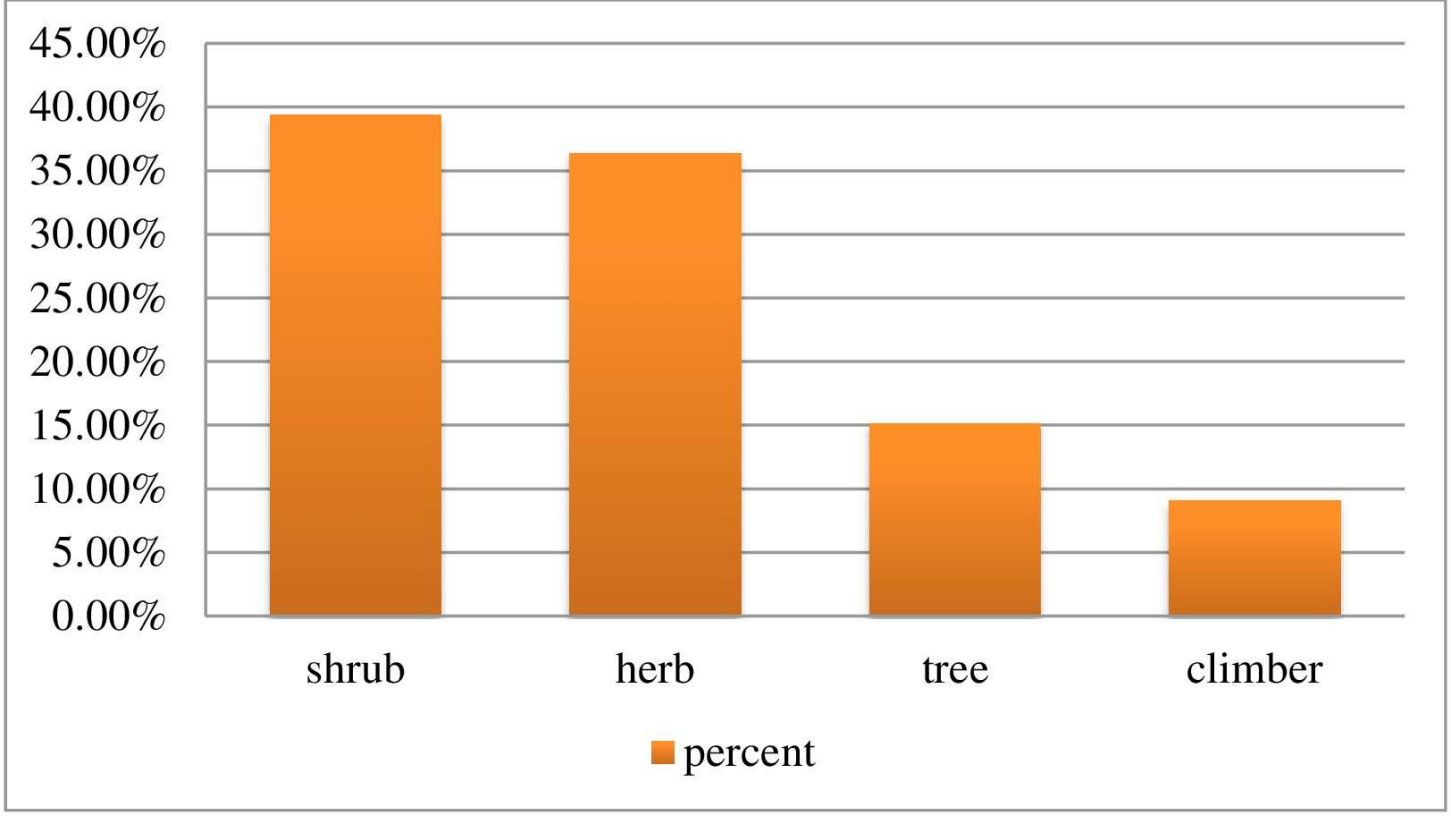
Growth forms of the plant.

Regarding growth forms, shrubs were the most harvested to treat livestock ailments and were represented with 13 (39.39%) plant species followed by herbs 12(36.36%) and trees 5(15.15%). On the other hand, climber was minor plant habit 3 (9.09%) used to treat livestock ailments (Fig 2). From the total of 33 medicinal plants reported, 26 (78.79%) were obtained from the wild, 6 (18.18%) from the home garden, and the remaining single (3.03%) plant species were obtained from both wild and cultivated (Fig 2).

#### Types of livestock ailments in the study district

In this study, a total of 18 types of livestock ailments were identified. The practitioners reported that they might use one or more medicinal plant species to treat specific type of ailment. Of those ailments, blackleg was the most common type of ailments and treated by 10 medicinal plant species (Table 2).

**Table 2:** Informant Consensus Factor (ICF) of different ailments.

Types of ailments	No plants used	% of the species	number of use citation	% of citation	ICF value
Eye defect	2	6.06	4	2.53	0.67
Diarrhea	3	9.09	8	5.06	0.71
New castle	2	6.06	5	3.16	0.75
Blackleg	10	30.3	51	32.28	0.82
Colic	2	6.06	4	2.53	0.67
Tania	4	12.12	12	7.59	0.73
Listeriosis	2	6.06	5	3.16	0.75
Leech	2	6.06	3	1.9	0.5
Pasturalosis	4	12.12	15	9.49	0.79
Mite infestation	4	12.12	9	5.7	0.63
Myiasis	2	6.06	4	2.53	0.67
General illness	7	21.21	31	19.62	0.8
Adnominal pain	2	6.06	3	1.9	0.5
Footrot	2	6.06	4	2.53	0.67

ICF value of this table the same as ISSN 2224-3208 (Paper) ISSN 2225-093X (Online).

#### Plant parts used for preparation of the remedies

In the study district, the most widespread plant parts used to prepare remedies was leaf (55.36%) followed by root (23.21%) and seed (8.93%) respectively. The remaining bark, all parts of the plants, sap, and bulb contributed for 12.5% of remedies preparation. Regarding the condition of the plant parts, freshly harvested plant parts were the dominant (52.94%) and the remaining 47.06% were used in the form of both freshly and in the dry form.

#### Mode of preparation and routs of administration

**Fig 3:**
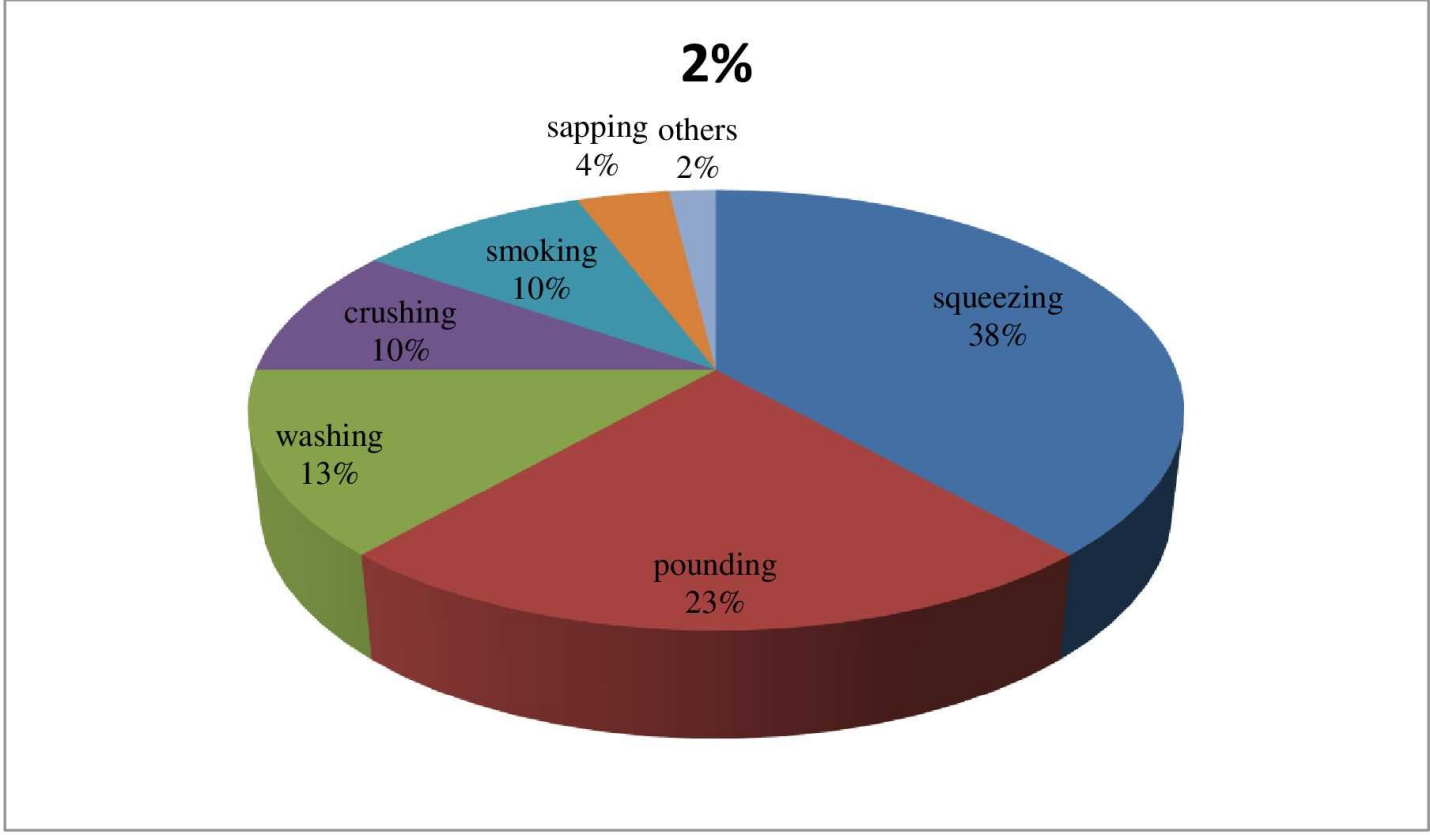
Mode of application of medicinal plants.

Various method of remedies preparation was applied by the practitioners depending on types of ailments. In this study the most common types of remedies preparation was squeezing (37.74%), followed by pounding (22.64%) and washing (13.21%). The next rank was taken by crushing and smoking (9.43%) each. Followed this sapping took the rank (3.77%). The remaining type of preparation (streaking and pasting) were covered 1.89% each. Regarding routes of administration oral was the leading (61.54%); followed by dermal (19.23%), nasal (15.38%), and optical (3.85%) Fig 3. S1 Table.

#### Marketability of medicinal plants

A Survey took place in five local markets of the district; Seladingaye 01, Sasit, Asofe, Begochgate, and Tarmaber. The results showed that, from the total of 33 medicinal plants recorded 13 (39.39%) plant species were marketable. Out of these, 13 marketable medicinal plants *Lipidium sativum*, *Silene macrosolen*, and *Echinops kebericho* were marketed totally for medicinal value, and the other 10 marketable plant species were sold and purchased for other purposes such as construction, spice, food and firewood.

#### Efficiency of ethnoveterinary medicinal plants

The highest informants’ consensus factor (ICF) values were recorded for Blackleg (0.82); followed by general illness (0.8) and pasturalosis (0.79). The next rank was taken by a new castle and Listeriosis (0.75) each. At the same time, the highest plant citation 10(30.3%) was recorded for blackleg and general illness 7(21.21%) Table 2.

#### Preference ranking

Based on the information obtained from 15 key informants, *Vernonia amygdalina* was the most effective medicinal plant to treat blackleg. The next most effective medicinal plants were *Solanecio gigas* and *Cucumis ficifolius*. The information obtained from fifteen key informants also showed that comparatively *Jasminum abyssinicum* and *Solanum incanum* were the least effective medicinal plants (Table 3).

**Table 3:** Preference ranking medicinal plants to treat blackleg.

Plant species	Respondents															Total	Rank
	R_1_	R_2_	R_3_	R_4_	R_5_	R_6_	R_7_	R_8_	R_9_	R_10_	R_11_	R_12_	R_13_	R_14_	R_15_		
*Cardiospermum corundum*	1	6	3	2	5	3	1	5	4	3	1	5	6	4	6	55	8^th^
*Cucumis ficifolius*	8	8	10	6	9	10	4	1	6	1	10	9	8	8	10	108	3^rd^
*Jasminum abyssinicum*	3	5	2	1	4	2	3	2	2	2	6	4	4	2	3	45	9^th^
*Lipidium sativum*	6	4	7	10	2	6	9	10	5	7	3	7	9	6	7	98	5^th^
*Ruta chalepensis*	7	9	6	8	8	7	6	4	7	9	5	6	10	7	5	14	4^th^
*Solanecio gigas*	10	3	9	7	10	9	2	8	9	10	9	8	7	10	9	10	2^nd^
*Phytolacca dodecandra*	4	7	5	3	6	5	8	3	1	5	2	3	2	5	4	63	7^th^
*Solanum incanum*	2	1	4	4	1	1	5	7	3	4	4	1	5	1	2	45	9^th^
*Verbascum sinaiticum*	5	2	1	5	3	4	7	9	8	6	8	2	1	3	1	65	6^th^
*Vernonia amygdalina*	9	10	8	9	7	8	10	6	10	8	7	10	3	9	8	12	1^st^

The reference this table the same as doi: 10.1186/1746-4269-10-21.

#### Threat for medicinal plants in the district

In the study district, the survival of medicinal plants affected by both natural (such as dry condition and landslide) and anthropogenic factors (firewood, overgrazing, agricultural expansion, construction, and medicinal usage) activities. Expansion of agriculture flowed by drought and construction were the major threats for medicinal plants in the study district (Table 4).

**Table 4:** Major threats for medicinal plants for study district.

Factors	Respondents															Total	Rank
	R_1_	R_2_	R_3_	R_4_	R_5_	R_6_	R_7_	R_8_	R_9_	R_10_	R_11_	R_12_	R_13_	R_14_	R_15_		
Drought	5	4	6	4	6	7	4	6	7	4	6	4	6	7	3	79	2^nd^
Landslide	2	3	2	3	3	4	2	4	5	3	4	2	7	3	1	48	6^th^
Firewood	6	7	5	5	2	3	3	5	1	1	2	6	3	4	5	58	4^th^
Overgrazing	4	5	1	1	4	5	5	1	2	5	3	3	2	2	6	49	5^th^
Agriculture	7	6	7	7	7	6	7	7	6	7	7	7	5	5	7	98	1^st^
Construction	3	2	4	6	5	2	6	3	4	6	5	5	4	6	4	65	3^rd^
Medicinal value	1	1	3	2	1	1	1	2	3	2	1	1	1	1	2	23	7^th^

The table of this reference of this table is the same as DOI 10.1186/s13002-018-0212-0.

## Discussion

In the study district, the livelihoods of the people depend on livestock for crop production, marketing purpose, and for their income. This makes the people of the district to have knowledge of medicinal plants to protect their livestock from different ailments; even though the knowledge was varied with age and sex group. In this study, most respondents (79.05%) were men, and there were very few women (20.95%). The cause for this might be due to the fact that the elders pass their knowledge secrecy either for their elder son or for their selected son rather than their daughter. Similar findings were reported in a different part of Ethiopia [19,20]. Even if men respondents reported more medicinal plants than women in average, statistically (p = 0.53) it is not significant. This finding was in line with the work of [21]. The study also showed that a significantly higher average number of medicinal plants (p <0.05) was reported by elder groups of informants than youngest group. The root cause for this significant difference is an expansion of modern medication, and lack of interest in the youngest age groups to traditional type of medication. In addition to this, unavailability of medicinal plants at every season, and being laborious to harvest cause for lack of interest in the youngest group. Thus, decreasing positive attitude towards traditional medicine is an indication of the erosion of knowledge of traditional medicine and medicinal plants species. The same finding was conducted in the work of [19,21]. Correspondingly, illiterate respondents reported significantly (p < 0.05) higher number of medicinal plants than educated respondents. This is due to the fact that educated respondents did not give attention to traditional medicine and prefer modern medicine. This in turn cause for degradation of medicinal knowledge in the coming generation. The same finding was reported by [20]. Due to the result of experience, key informants could report significant (p < 0.05) number of medicinal plants than general informants.

### Diversity of ethnoveterinary medicinal plants

Thirty -three ethnoveterinary medicinal plant species, which belong to 23 families and 31 genera were identified, and documented with their local name, scientific name, habit, mode of preparation, and used parts. Family Asteraceae and Solanaceae were dominant. The dominance of family Solanaceae was in line with other findings reported in different parts of Ethiopia [14,22–24], and dominancy of family Asteraceae was also reported in the district of Ankober, North Showa, Amhara region, Ethiopia and Kansas and the Great Plains [19]. From the total of 33 species, 81.82% was covered by endemic and indigenous plant species. This result clearly indicates that the district is rich in biodiversity. In addition to this preferring of endemic and indigenous plant species for medicinal purposes indicates that the knowledge of the people is not recent rather than it had a long history, and it passes from generation to generation for a long period of time. This finding was in line with the finding of [19,25].

The growth form analysis of medicinal plants revealed that shrubs were constituted the highest proportion being represented by 13 species (39.39%), followed by herbs which is represented by 12 species (36.36%) while trees and climber were represented by 5 (15.15%) and 3 (9.09%) plant species respectively. High usage of shrub for medicinal value was oobserved in a different part of Ethiopia [19,26,27]. This may be due to the relative abundance of shrub and availability of it to the practitioners in the study district. In contrast, other researchers reported that herbs are dominant [14,22,23].

In this study, most of the plants 26 (78.79%) were harvested from the wild and the others 6 (18.18%) were collected from the home garden and the remaining single (3.03%) species was available in both home gardens and wild. This finding was in line with the of [19,22,23,27]. This clearly indicates that in the study area the practice of cultivated plants in the home garden for medicinal purposes is very rare. This less habit of cultivation of medicinal plants in home gardens causes for unavailability of those medicinal plants as the practitioners want in all season of the year.

### Plant parts used for preparation of the remedies

Different parts of the plants have been used to cure different ailments; however, leaves (55.3%) were the most widely used part of the plants in the study district followed by roots (23.21%). This finding agrees with works of [28] in Amaro special districts of southern Ethiopia [22], in Horro Gudurru district of western Ethiopia, [14] in Melkabello district of eastern Harerghe; however, it contradicts with the works of [29] in Berbere District of Bale zone of Oromia region and [23] at Enarj Enawega district of east Gojjam zone of Amhara region. Usage of leaves for medicine has less effect on the survival of the plants in their natural habitat. However, usage of roots for medicinal value would cause for extinction of the plants from their natural habitat and also might cause for erosion of medicinal plant knowledge in the district. Most of the practitioners in the study district prefer the plants in fresh (52.94%) conditions. This finding agrees with the findings of [30]. Collection of fresh plant material might cause for divesting of the plant species when the practitioners prepare remedies during the dry season.

### Mode of preparation and routs of administration

The result showed that squeezing method of remedies preparation was dominant in the study District, followed by pounding and washing. This finding was similar with the work of [25]. Regarding on mode of administration, oral mode of administration was the most dominant and accounting for 61.54% followed by dermal (19.23%) and nasal (15.38%). This finding was with the finding of [21]. In the study district, there is no standardized known unit to measure the plant remedies rather they use their own measurement such as ATSEKE, SHEKENA, SENZERE, TASA. However, the dosage of the remedies depends on the age, size, and type of livestock which are treated. The same result was reported by [24,31,32].

### Efficiency of ethnoveterinary medicinal plants

The highest informants’ consensus factor (ICF) values were recorded for Blackleg (0.82), general illness (0.8), and pasturalosis (0.79). At the same time, the highest plant citation 10(30.3%) was recorded for blackleg, followed by general illness 7(21.21%). This clearly indicate that blackleg is a common and well known ailment for the study district. To treat this ailment, 10 ethnoveterinary medicinal plants were reported. Of which medicinal plants, *Vernonia amygdalina* was the most preferred plant species; followed by *Solanecio gigas* and *Cucumis ficifolius* to heal blackleg. Some of the ethnoveterinary medicinal plants which were listed in the study districts to heal blackleg were used for the same ailment in different districts [32,33].

Generally, the study district is rich in biodiversity, and the people have extensive indigenous knowledge about ethnoveternary medicinal plants which is important to treat various types of livestock ailments. As well the study area is rich in endemic species. The result of this study showed that the knowledge and medicinal plants of the district are at the risk. So, it requires special attention from the government, people and all stakeholders.

## Supporting information

S1 Table Name of informants with their sex, age, educational level and no of plants cited by them. Note R represent respondents which were participated during data collection time.
(DOCX)

S2 Table List of ethnoveterinary medicinal plants: Scientific name; family name; local name; habit; Source of the; plant parts used; animal treated; ailment treated; preparation and application; condition of the plants used and route of administration. (DOCX)

S1 Appendix Amharic and English version of questioner. The Amharic version of questioner was given to the informants to collect data.
(DOCX)

S2 Appendix Sample of informant’s response for the questioner. (DOCX)

## References

[1] Eshetie Tekeba, Hussien Kelifa, Teshome Tadesse, et al. Meat production, consumption and marketing tradeoffs and potentials in Ethiopia and its effect on GDP growth: a review. *J Nutr Health Food Eng*, (2018) 8(3), 228–233.

[2] Asresie A., Zemedu L., & Adigrat E. The contribution of livestock sector in Ethiopian economy. *A Review Advances in Life Science And Technology*, (2015) 29.

[3] Negassa A., Rashid S., Gebremedhin B., & Kennedy A. Livestock production and marketing. *Food and agriculture in Ethiopia*: *Progress and policy challenges*, (2012) 159–189.

[4] Hoddinott J., Headey D., & Dereje M. Cows, missing milk markets, and nutrition in rural Ethiopia. *The Journal of Development Studies*, (2015) 51(8), 958–975.

[5] CSA (Central Statistical Authority). Livestock sample survey Central Statistical Agency, Ministry of Finance and Economic Development. Addis Ababa, Ethiopia. (2011–12).

[6] Duguma B. Smallholder livestock production and marketing systems in the Haramaya District, Eastern Ethiopia. Basic Res J Agric Sci Rev. (2013) 2:122–129.

[7] Gurib-Fakim A, Brendler T, Philips, L.D, and Eloff, J.N. Traditional Medicines in Africa: An Appraisal of Ten Potent African Medicinal Plants. In Evidence-based Complementary and Alternative Medicine. (2013). 10.1155/2013/617459 PMC3866779 24367388

[8] Bekele D., Asfaw Z., Petros B., & Tekie H. Ethnobotanical study of plants used for protection against insect bite and for the treatment of livestock health problems in rural areas of Akaki District, Eastern Shewa, Ethiopia. *Topclass Journal of Herbal Medicine*, (2012). 1(2), 12–24.

[9] Gebreyes W. A., Dupouy-Camet J., Newport M. J., Oliveira C. J., Schlesinger L. S., Saif Y. M., et al. The global one health paradigm: challenges and opportunities for tackling infectious diseases at the human, animal, and environment interface in low-resource settings. *PLoS Negl Trop Dis*, (2014) 8(11), e3257. doi: 10.1371/journal.pntd.0003257 25393303 PMC4230840

[10] Aziz M. A., Khan A. H., Adnan M., & Ullah H. Traditional uses of medicinal plants used by Indigenous communities for veterinary practices at Bajaur Agency, Pakistan. *Journal of ethnobiology and ethnomedicine*, (2018). 14(1), 1–18. 29378636 10.1186/s13002-018-0212-0 PMC5789696

[11] Gonfa N., Tulu D., Hundera K., & Raga D. Ethnobotanical study of medicinal plants, its utilization, and conservation by indigenous people of Gera district, Ethiopia. *Cogent Food & Agriculture*, (2020) 6(1), 1852716.

[12] Taha E., & Woldeyohannes M. S. Herbalists and their Mode of Health Care Service Delivery in Debre Markos Town, Northwest Ethiopia. *Advanced Journal of Social Science*, (2020) 6(1), 122–137.

[13] Rahmawati N., Mustofa F. I., Haryanti S., & Mujahid R. Medicinal plant utilization for hypercholesterolemia by traditional healers in Java Island. In *IOP Conference Series*: *Earth and Environmental Science* (2021, January). (Vol. 637, No. 1, p. 012043). IOP Publishing.

[14] Mohammed C., Abera D., Woyessa M., & Birhanu T. Survey of ethno-veterinary medicinal plants in Melkabello District, eastern Harerghe zone, Eastern Ethiopia. *Ethiopian Veterinary Journal*, (2016) 20(2), 1–15.

[15] Cotton C.M. Ethnobotany: Principles and Applications. John Wiley and Sons, New York, 412pp. (1996).

[16] Abdisa and Tagesu. Mechanism of retained placenta and its treatment by plant medicine in ruminant animals in Oromia, Ethiopia. *Journal of Veterinary Medicine and Animal Health*, (2018). 10(6), 135–147.

[17] Teklay Abraha, Abera Balcha, Giday Mirutse. An ethnobotanical study of medicinal plants used in Kilte Awulaelo District, Tigray Region of Ethiopia. *Journal of ethnobiology and ethnomedicine*, (2013) 9(1), 1–23. doi: 10.1186/1746-4269-9-65 24011232 PMC3852788

[18] Chekole Getnet. Ethnobotanical study of medicinal plants used against human ailments in Gubalafto District, Northern Ethiopia. *Journal of ethnobiology and ethnomedicine*, (2017) 13(1), 1–29. 28978342 10.1186/s13002-017-0182-7 PMC5628421

[19] Lulekal E, Asfaw Z, Kelbessa E, Damme PV. Ethnoveterinary plants of Ankober districts, North Shewa zone, Amhara region, Ethiopia. *Journal of Ethnobiology and Ethnomedicine*. (2014). 10.1186/1746-4269-10-21 PMC3924401 24517385

[20] Birhan YS, Kitaw SL, Alemayehu YA and Mengesha NM. Ethnoveterinary Medicinal Plants and Practices in Enarj Enawga District, East Gojjam Zone, Amhara Region, Ethiopia. *Int J Anim Sci*. (2018) 2(1): 1014.

[21] Yigezu Y, Haile DB, Ayen WA. Ethnoveterinary medicine in four districts of Jimma zone, Ethiopia: cross sectional survey for plant species and mode of use. *BMC veterinary research*, (2014) 10:76. doi: 10.1186/1746-6148-10-76 24679045 PMC3978085

[22] Birhanu T., & Abera D. Survey of ethno-veterinary medicinal plants at selected Horro Gudurru Districts, Western Ethiopia. *African Journal of Plant Science*, (2015) 9(3), 185–192.

[23] Seid M. Critical solutions for critical problems: Threats to sustainable use and management of Nech Sar National Park (NSNP) in Ethiopia. Afr J Hosp Tour Leis (2019) 8: 1. doi: 10.20546/ijcrar.701.002

[24] Kidane Leul, Gebremedhin Gebrecherkos, Beyene Tadesse. Ethnobotanical study of medicinal plants in Ganta Afeshum District, Eastern Zone of Tigray, Northern Ethiopia. *Journal of ethnobiology and ethnomedicine*, (2018) 14(1), 1–19. 30390675 10.1186/s13002-018-0266-z PMC6215673

[25] Mengesha, Getaneh Gebeyehu. Ethnobotanical survey of medicinal plants used in treating human and livestock health problems in Mandura Woreda of Benishangul Gumuz, Ethiopia. *Adv Med Plant Res*, (2016) 4(1), 11–26.

[26] Giday M., & Teklehaymanot T. Ethnobotanical study of plants used in management of livestock health problems by Afar people of Ada’ar District, Afar Regional State, Ethiopia. *Journal of Ethnobiology and Ethnomedicine*, (2013) 9(1), 1–10. doi: 10.1186/1746-4269-9-8 23343251 PMC3561197

[27] Usmane A., Birhanu T., Redwan M., Sado E., & Abera D. Survey of ethno-veterinary medicinal plants at selected districts of Harari Regional State, Eastern Ethiopia. *Ethiopian Veterinary Journal*, (2016) 20(1), 1–22.

[28] Tekle Y. Study on ethno veterinary practices in Amaro special district southern Ethiopia. *Med Aromat Plants*, (2015) 4(186), 2167–0412.

[29] Jima T. T., & Megersa M. Ethnobotanical study of medicinal plants used to treat human diseases in Berbere District, Bale Zone of Oromia Regional State, South East Ethiopia. *Evidence-Based Complementary and Alternative Medicine*, 2018. doi: 10.1155/2018/8602945 30105073 PMC6076952

[30] Chekole G., Asfaw Z., & Kelbessa E. Ethnobotanical study of medicinal plants in the environs of Tara-gedam and Amba remnant forests of Libo Kemkem District, northwest Ethiopia. *Journal of ethnobiology and ethnomedicine*, (2015) 11(1), 1–38. doi: 10.1186/1746-4269-11-4 25572933 PMC4417315

[31] Tolossa K., Debela E., Athanasiadou S., Tolera A., Ganga G., & Houdijk J. G. Ethno-medicinal study of plants used for treatment of human and livestock ailments by traditional healers in South Omo, Southern Ethiopia. *Journal of Ethnobiology and Ethnomedicine*, (2013) 9(1), 1–15. doi: 10.1186/1746-4269-9-32 23680260 PMC3679849

[32] Ayehu M., & Debebe D. Ethno veterinary medicine knowledge and practices in and around gondar, Ethiopia. *International Journal of Public Health*, *Pharmacy and Pharmacology*, (2018).3(1), 39–68.

[33] Tadesse B., Mulugeta G., Fikadu G., Sultan A., & Nekemte E. Survey on ethno-veterinary medicinal plants in selected Woredas of east Wollega zone, western Ethiopia. *Journal of Biology*, *Agriculture and Healthcare*, (2014) 4(17), 97–105.

