# Supplementary material for: The study of ethnoveterinary medicinal plants at Mojana Wodera district, central Ethiopia
Source: PLoS One. 2022 May 25;17(5):e0267447. doi: 10.1371/journal.pone.0267447 (PMC9132277; doi:10.1371/journal.pone.0267447)
Supplement: S1 Table — Note R represent respondents which were participated during data collection time. (DOCX) [file pone.0267447.s001.docx]

| \| Name of informants in their code \| Sex \| Age \| Educational level \| No of plants cited by them \| \| --- \| --- \| --- \| --- \| --- \| \| R 1 \| Female \| 20-39 \| llterate \| 1 \| \| R 2 \| male \| 20-39 \| llterate \| 3 \| \| R 3 \| Female \| 40-85 \| illterate \| 3 \| \| R 4 \| male \| 40-85 \| illterate \| 5 \| \| R 5 \| Female \| 40-85 \| llterate \| 4 \| \| R 6 \| male \| 40-85 \| llterate \| 1 \| \| R 7 \| male \| 20-39 \| llterate \| 1 \| \| R 8 \| male \| 40-85 \| llterate \| 2 \| \| R 9 \| Female \| 40-85 \| illterate \| 3 \| \| R 10 \| male \| 40-85 \| llterate \| 6 \| \| R 11 \| male \| 20-39 \| llterate \| 3 \| \| R 12 \| male \| 40-85 \| llterate \| 6 \| \| R 13 \| male \| 40-85 \| illterate \| 2 \| \| R 14 \| male \| 20-39 \| llterate \| 1 \| \| R 15 \| male \| 40-85 \| illterate \| 3 \| \| R 16 \| Female \| 40-85 \| llterate \| 3 \| \| R 17 \| Female \| 20-39 \| llterate \| 2 \| \| R 18 \| male \| 40-85 \| illterate \| 5 \| \| R 19 \| male \| 40-85 \| llterate \| 5 \| \| R 20 \| male \| 40-85 \| llterate \| 4 \| \| R 21 \| male \| 20-39 \| llterate \| 3 \| \| R 22 \| male \| 40-85 \| illterate \| 3 \| \| R 23 \| Female \| 40-85 \| illterate \| 1 \| \| R 24 \| male \| 20-39 \| llterate \| 1 \| \| R 25 \| male \| 40-85 \| illterate \| 4 \| \| R 26 \| male \| 40-85 \| llterate \| 2 \| \| R 27 \| male \| 20-39 \| llterate \| 2 \| \| R 28 \| male \| 40-85 \| illterate \| 1 \| \| R 29 \| male \| 40-85 \| illterate \| 3 \| \| R 30 \| Female \| 40-85 \| llterate \| 4 \| \| R 31 \| male \| 20-39 \| llterate \| 2 \| \| R 32 \| Female \| 40-85 \| llterate \| 3 \| \| R 33 \| male \| 40-85 \| llterate \| 5 \| \| R 34 \| male \| 40-85 \| llterate \| 1 \| \| R 35 \| male \| 40-85 \| illterate \| 3 \| \| R 36 \| male \| 40-85 \| llterate \| 2 \| \| R 37 \| male \| 20-39 \| llterate \| 1 \| \| R 38 \| male \| 40-85 \| illterate \| 3 \| \| R 39 \| male \| 40-85 \| llterate \| 2 \| \| R 40 \| male \| 20-39 \| llterate \| 3 \| \| R 41 \| male \| 40-85 \| llterate \| 3 \| \| R 42 \| male \| 40-85 \| illterate \| 2 \| \| R 43 \| male \| 40-85 \| llterate \| 2 \| \| R 44 \| male \| 20-39 \| llterate \| 1 \| \| R 45 \| Female \| 40-85 \| illterate \| 5 \| \| R 46 \| male \| 40-85 \| llterate \| 3 \| \| R 47 \| male \| 40-85 \| llterate \| 3 \| \| R 48 \| male \| 20-39 \| illterate \| 3 \| \| R 49 \| Female \| 40-85 \| llterate \| 1 \| \| R 50 \| Female \| 40-85 \| llterate \| 1 \| \| R 51 \| Female \| 40-85 \| illterate \| 1 \| \| R 52 \| male \| 20-39 \| llterate \| 3 \| \| R 53 \| Female \| 40-85 \| illterate \| 2 \| \| R 54 \| Female \| 20-39 \| llterate \| 1 \| \| R 55 \| male \| 40-85 \| illterate \| 4 \| \| R 56 \| male \| 20-39 \| llterate \| 2 \| \| R 57 \| male \| 40-85 \| llterate \| 1 \| \| R 58 \| male \| 20-39 \| llterate \| 2 \| \| R 59 \| male \| 40-85 \| illterate \| 3 \| \| R 60 \| Female \| 20-39 \| llterate \| 1 \| \| R 61 \| male \| 40-85 \| illterate \| 4 \| \| R 62 \| male \| 20-39 \| llterate \| 1 \| \| R 63 \| male \| 40-85 \| illterate \| 3 \| \| R 64 \| male \| 40-85 \| illterate \| 3 \| \| R 65 \| male \| 20-39 \| llterate \| 1 \| \| R 66 \| male \| 40-85 \| illterate \| 1 \| \| R 67 \| male \| 40-85 \| llterate \| 7 \| \| R 68 \| Female \| 20-39 \| llterate \| 1 \| \| R 69 \| male \| 40-85 \| illterate \| 3 \| \| R 70 \| Female \| 40-85 \| llterate \| 1 \| \| R 71 \| Female \| 20-39 \| llterate \| 1 \| \| R 72 \| male \| 40-85 \| illterate \| 5 \| \| R 73 \| male \| 40-85 \| illterate \| 4 \| \| R 74 \| male \| 40-85 \| illterate \| 4 \| \| R 75 \| male \| 40-85 \| llterate \| 5 \| \| R 76 \| Female \| 20-39 \| llterate \| 2 \| \| R 77 \| male \| 40-85 \| illterate \| 3 \| \| R 78 \| male \| 20-39 \| llterate \| 1 \| \| R 79 \| male \| 40-85 \| illterate \| 3 \| \| R 80 \| male \| 40-85 \| illterate \| 4 \| \| R 81 \| Female \| 40-85 \| illterate \| 2 \| \| R 82 \| male \| 20-39 \| llterate \| 1 \| \| R 83 \| male \| 40-85 \| illterate \| 3 \| \| R 84 \| male \| 20-39 \| llterate \| 1 \| \| R 85 \| male \| 40-85 \| illterate \| 2 \| \| R 86 \| male \| 20-39 \| llterate \| 1 \| \| R 87 \| male \| 40-85 \| llterate \| 4 \| \| R 88 \| male \| 20-39 \| llterate \| 1 \| \| R 89 \| male \| 40-85 \| illterate \| 3 \| \| R 90 \| male \| 20-39 \| llterate \| 1 \| \| R 91 \| male \| 40-85 \| illterate \| 2 \| \| R 92 \| male \| 20-39 \| llterate \| 1 \| \| R 93 \| male \| 40-85 \| llterate \| 1 \| \| R 94 \| male \| 20-39 \| illterate \| 2 \| \| R 95 \| male \| 40-85 \| llterate \| 2 \| \| R 96 \| male \| 40-85 \| illterate \| 2 \| \| R 97 \| male \| 20-39 \| llterate \| 1 \| \| R 98 \| male \| 40-85 \| illterate \| 2 \| \| R 99 \| Female \| 20-39 \| llterate \| 1 \| \| R 100 \| male \| 40-85 \| llterate \| 1 \| \| R 101 \| male \| 20-39 \| llterate \| 1 \| \| R 102 \| male \| 40-85 \| llterate \| 1 \| \| R 103 \| male \| 20-39 \| illterate \| 1 \| \| R 104 \| male \| 40-85 \| illterate \| 2 \| \| R 105 \| male \| 20-39 \| llterate \| 2 \| |
| --- | --- | --- | --- | --- | --- | --- | --- | --- | --- | --- | --- | --- | --- | --- | --- | --- | --- | --- | --- | --- | --- | --- | --- | --- | --- | --- | --- | --- | --- | --- | --- | --- | --- | --- | --- | --- | --- | --- | --- | --- | --- | --- | --- | --- | --- | --- | --- | --- | --- | --- | --- | --- | --- | --- | --- | --- | --- | --- | --- | --- | --- | --- | --- | --- | --- | --- | --- | --- | --- | --- | --- | --- | --- | --- | --- | --- | --- | --- | --- | --- | --- | --- | --- | --- | --- | --- | --- | --- | --- | --- | --- | --- | --- | --- | --- | --- | --- | --- | --- | --- | --- | --- | --- | --- | --- | --- | --- | --- | --- | --- | --- | --- | --- | --- | --- | --- | --- | --- | --- | --- | --- | --- | --- | --- | --- | --- | --- | --- | --- | --- | --- | --- | --- | --- | --- | --- | --- | --- | --- | --- | --- | --- | --- | --- | --- | --- | --- | --- | --- | --- | --- | --- | --- | --- | --- | --- | --- | --- | --- | --- | --- | --- | --- | --- | --- | --- | --- | --- | --- | --- | --- | --- | --- | --- | --- | --- | --- | --- | --- | --- | --- | --- | --- | --- | --- | --- | --- | --- | --- | --- | --- | --- | --- | --- | --- | --- | --- | --- | --- | --- | --- | --- | --- | --- | --- | --- | --- | --- | --- | --- | --- | --- | --- | --- | --- | --- | --- | --- | --- | --- | --- | --- | --- | --- | --- | --- | --- | --- | --- | --- | --- | --- | --- | --- | --- | --- | --- | --- | --- | --- | --- | --- | --- | --- | --- | --- | --- | --- | --- | --- | --- | --- | --- | --- | --- | --- | --- | --- | --- | --- | --- | --- | --- | --- | --- | --- | --- | --- | --- | --- | --- | --- | --- | --- | --- | --- | --- | --- | --- | --- | --- | --- | --- | --- | --- | --- | --- | --- | --- | --- | --- | --- | --- | --- | --- | --- | --- | --- | --- | --- | --- | --- | --- | --- | --- | --- | --- | --- | --- | --- | --- | --- | --- | --- | --- | --- | --- | --- | --- | --- | --- | --- | --- | --- | --- | --- | --- | --- | --- | --- | --- | --- | --- | --- | --- | --- | --- | --- | --- | --- | --- | --- | --- | --- | --- | --- | --- | --- | --- | --- | --- | --- | --- | --- | --- | --- | --- | --- | --- | --- | --- | --- | --- | --- | --- | --- | --- | --- | --- | --- | --- | --- | --- | --- | --- | --- | --- | --- | --- | --- | --- | --- | --- | --- | --- | --- | --- | --- | --- | --- | --- | --- | --- | --- | --- | --- | --- | --- | --- | --- | --- | --- | --- | --- | --- | --- | --- | --- | --- | --- | --- | --- | --- | --- | --- | --- | --- | --- | --- | --- | --- | --- | --- | --- | --- | --- | --- | --- | --- | --- | --- | --- | --- | --- | --- | --- | --- | --- | --- | --- | --- | --- | --- | --- | --- | --- | --- | --- | --- | --- | --- | --- | --- | --- | --- | --- | --- | --- | --- | --- | --- | --- | --- | --- | --- | --- | --- | --- | --- | --- | --- | --- | --- | --- | --- | --- | --- | --- | --- | --- | --- | --- | --- | --- | --- | --- | --- | --- | --- | --- | --- | --- | --- | --- | --- | --- | --- | --- | --- | --- | --- | --- | --- | --- | --- | --- | --- | --- | --- | --- | --- | --- | --- | --- | --- | --- | --- | --- | --- | --- | --- | --- | --- | --- | --- | --- | --- | --- | --- | --- |

R: refers to name of respondents
