# Supplementary material for: The study of ethnoveterinary medicinal plants at Mojana Wodera district, central Ethiopia
Source: PLoS One. 2022 May 25;17(5):e0267447. doi: 10.1371/journal.pone.0267447 (PMC9132277; doi:10.1371/journal.pone.0267447)
Supplement: S2 Table — (DOCX) [file pone.0267447.s002.docx]

**Table 5. List of ethnoveterinary medicinal plants: Scientific name; family name; local name; habit; Source of the; plant parts used; animal treated; ailment treated; preparation and application; condition of the plants used and route of administration.**

| V. No | Scientific name | Family name | Local name | habit | SOP | Parts | Animal treated | Ailment treated | PMAP | CPU | RUA |
| --- | --- | --- | --- | --- | --- | --- | --- | --- | --- | --- | --- |
| M3 | *Allium sativum* L. @ | Alliaceae | Nechi shenkurt | H | HG | Bu | Cattle | General illness | 1 | F/D | O |
| M9 | *Aloe genus* # | Aloaceae | Wende rate | S | W | Sa | Hen | New castle | 2 | F | O |
| M12 | *Aloe pulcherrima* Gilbert & Sebsebe * | Aloaceae | Sete rate | S | W | Sa | Hen | New castle | 2 | F | O |
| M15 | *Artemisia afra* Jacq. ex Willd. # | Asteraceae | Chekugne | H | W | L | Cattle | Diarrhea | 3 | F | O |
| M20 | *Buddleja polystachya* Fresen. # | Loganiaceae | anefare | T | W | R, L | Cattle | Diarrhea | 7 | F/D | N |
| M23 | *Calpurnia aurea* (Ait.) Benth # | Fabaceae | Digita | S | W | L | Cattle | Mite infestation | 6 | F | De |
| M10 | *Cardiospermum corundum* L.*#* | Sapindaceaee | smeg | C | W | R | Cattle | Black leg | 1 | F/D | O |
| M1 | *Croton macrostachyus* Del. # | Euphorbiaceae | Besana | T | W | L | Goat, sheep and cattle | Foot rot | 6 | F | De |
|  |  |  |  |  |  |  |  | Trauma | 1 | F | De |
| M14 | *Cucumis ficifolius* A. Rich # | Cucurbitaceae | Ymider enboye | H | W | R | Cattle | Black leg | 1 | F/D | O |
| M32 | *Datura stramonium* L. @ | Solanaceae | atefarise | H | W | L | Cattle | Myiasis | 8 | F | De |
| M21 | *Diplolophium africanum* Turcz. *#* | Apiaceae | Yeferse zenge | H | W | L | Cattle | Tania | 3 | F/D | O |
| M16 | *Echinops kebericho* Mesfin *** | Asteraceae | Kebercho | H | W | Al | Cattle | General illness | 7 | F/D | N |
| M2 | *Eucalyptus camaldulensis* Dehnh. *@* | Myrtaceae | Keye baherzafe | T | HG | L, Br | Cattle | General illness | 7 | F | N |
| M11 | *Eucalyptus globules* Labill @ | Myrtaceae | Neche baherzafe | T | HG | L, Br | Cattle | General illness | 7 | F | N |
| M22 | *Jasminum abyssinicum* Hochets. Ex DC. *#* | Oleaceae | Tero hareg/tenbelele | C | W | L | Cattle | Black leg | 3 | F | O |
| M24 | *Justicia schimperiana* (Hochest. Ex Nees) # | Acanthaceae | Sensel/ semiza | S | W | L | Dog, cat | Rabbis | 3 | F | O |
| M13 | *Lipidium sativum* L.*@* | Brassicaceae | feto | H | HG | Se | Cattle | Black leg | 4 | D | O |
|  |  |  |  |  |  | Se |  | Pasturalosis | 4 | D | O |
|  |  |  |  |  |  | Se |  | General illness | 4 | D | O |
| M26 | *Lonchocarpus laxiflorus* Guill. & Perr. *#* | Fabaceae | amera | T | W | R,L | Goats | Listeriosis | 1 | F/D | O |
| M28 | *Mukia maderaspatana* (L.) M.J. Roem. *#* | Cucurbitaceae | Yeamora misa | C | W | L | Cattle | Mite infestation | 6 | F | De |
| M31 | *Nicotiana tabacum* L @ | Solanaceae | tenbaho | H | HG | L | Cattle | Leech | 3 | F | N |
| M8 | *Ocimum lamiifolium* Hochst. Ex Benth # | Lamiaceae | damakese | S | WH | L | Cattle | Pasturalosis | 3 | F | O, N |
| M18 | *Pentas schimperiana* A. Rich. *#* | Rubiaceae | wyenagefte | S | W | R | Goat, sheep, cattle, equine | Eye defect | 3 | F/D | Op |
| M29 | *Phytolacca dodecandra* L. Herit. # | Phytolaccaceae | endode | S | W | L | Cattle | Leech | 3 | F | N |
|  |  |  |  |  |  | L |  | Black leg | 3 | F | O |
| M27 | *Rumex nepalensis* spreng # | Polygonaceae | tulte | H | W | R | Cattle, Sheep, goat | Abdominal pain | 1 | F/D | O |
|  |  |  |  |  |  | L | Cattle, Sheep, goat, Equine | Foot rot | 5 | F | De |
|  |  |  |  |  |  | R | Equine | Colic | 1 | F /D | O |
| M19 | *Ruta chalepensis* L. # | Rutaceae | Tena adame | S | HG | Se | Cattle | Black leg | 4 | F/D | O |
|  |  |  |  |  |  | Se |  | General illness | 4 | F/D | O |
| M30 | *Sansevieria ehrenbergii* Schweinf. Ex Baker # | Dracaenaceae | Wende kacha | S | W | L | Cattle | Mite infestation | 3 | F | De |
| M33 | *Silene macrosolen* A. Rich. *#* | Caryophyllaceae | Wegert | H | W | Al | Cattle | General illness | 7 | F/D | N |
| M25 | *Solanecio gigas* (Vatke) C. Jeffrey * | Asteraceae | Yeshekoko gomen | S | W | L | Cattle | Tania | 3 | F | O |
|  |  |  |  |  |  | L |  | Black leg | 3 | F/D | O |
|  |  |  |  |  |  | L |  | Diarrhea | 3 | F/D | O |
| M6 | *Solanum incanum* L. # | Solanaceae | enbuoye | S | W | L | Cattle | Eye defect | 3 | F | Op |
| M17 | *Urtica simensis* L.* | Urticaceae | sama | H | W | L | Cattle | Black leg | 3 | F | O |
|  |  |  |  |  |  | L |  | Pasturalosis | 3 | F | O |
| M5 | *Verbascum sinaiticum* Benth. # | Scrophulariaceae | Yeaheya joro | H | W | L | Cattle | Myiasis | 3 | F | De |
|  |  |  |  |  |  | R |  | Erection of hair | 1 | F/D | O |
|  |  |  |  |  |  | R |  | Abdominal pain | 1 | F/D | O |
|  |  |  |  |  |  | R, L |  | Black leg | 1,6 | F/D | O |
|  |  |  |  |  |  | R |  | Colic | 1 | F/D | O |
| M7 | *Vernonia amygdalina* Del. # | Asteraceae | gerawa | S | W | L, R | Cattle | Tania | 1,3 | F/D | O |
|  |  |  |  |  |  | L |  | Mange | 6 | F | De |
|  |  |  |  |  |  | L |  | Mite infestation | 6 | F | De |
|  |  |  |  |  |  | L |  | Black leg | 3 | F | O |
|  |  |  |  |  |  | L |  | Pasturalosis | 3 | F | O |
| M4 | *Withania somnifera* (L.) Dun. # | Solanaceae | gizewa | S | W | R | Cattle | Tania | 1 | F/D | O |

This table the same as <http://www.ethnobiomed.com/content/10/1/21>.

Key: for Ethiopia (endemic (*), indigenous (#), introduced (@). growth form (tree (t), shrub (S), herb (H), climber(C). Source of the plant (SOP), home garden (HG), wild (W), from home and garden (WH). Plant parts used (leaf (L), seed (Se), root (R), all parts (Al), bulb (Bu), sap (Sa), bark (Br). Preparation and mode of application (Pound the root or bulb and homogenized with water then give the liquid (1),The sap extract from the plant and give it with injera (2), Squeeze the leaf and extract the liquid part and homogenized with water (3), Crushed the fruit and mix the powder with water and make a liquid(4), Insert the leaf in hot water then streak the affected part (5), Wash the infected part with leaf (6), Leaf, bark, stem or root burned on fire and the smoke inhaled (7), Grind the leaf then pasted the crushed the plant part (8). Condition of plant used (CPU) fresh (F), dry (D). Route of administration (RUA) oral (O), dermal (De), nasal (N), optical (Op).
