# Supplementary material for: The study of ethnoveterinary medicinal plants at Mojana Wodera district, central Ethiopia
Source: PLoS One. 2022 May 25;17(5):e0267447. doi: 10.1371/journal.pone.0267447 (PMC9132277; doi:10.1371/journal.pone.0267447)
Supplement: S1 Appendix — The Amharic version of questioner was given to the informants to collect data. (DOCX) [file pone.0267447.s003.docx]

S1 Appendix: Amharic and English version of questioner

**የአማርኛ መጠይቅ**

ይህ ቃለ መጠይቅ የአከባቢው ማህበረሰብ በአከባቢው የሚከሰቱ የእንስሳት በሽታወችን እንዴት አደርጎ እነደሚከላከላቸውና ምን ዓይነት ዕፅዋትን እንደሚጠቀም እነዲሁም ለእፅዋቱ ያለውን እንክብካቤ ለመረዳትና ወደፊት ይኸንን እውቀት እንዴት ማስቀጠል እነደሚቻል ለመወያየት ስለሆነ እናነተም ይህንን ተገንዝባችሁ ቃለ መጠይቁን ተሞሉ ዘነድ በትህትና እጠይቃችኋለሁ፡፡ ስለ ትብብራችሁ አመሰግናለሁ፡፡

ግላዊ መረጃ

1. ስም________________________ ጾታ___ እድሜ _________ቀበሌ_______

**ሃይማኖት ፡-** ኦርቶዶክስ ሙስሊም ፕሮቴስታንት ሌላ

**የጋብቻ ሁኔታ፡-** ያገባ ያላገባ የፈታ

የትምህርት ደረጃ፡-

ትምህርት የሌለው መደበኛ ያልሆነ 1-8 ክፍል 9-12 ክፍል ድፕሎማ ድግሪ

1. ጤናበተመለከተ
   1. በአከባቢህ የሚከሰት የእንስሳት በሽታዓይነት አለ?___________ካለ የበሽታውን ዓይነት እና የምትጠቀመውን እፅዋት በሚከተለው ሰንጠረዥ ውስጥ አስቀመጥ ፡፡

| ተ.ቁ | የበሽታው ዓይነት | መተላለፊያ መንገዱ | በሽታውን ለማዳን የምትጠቀመው የእፅዋት ዓይነት | የምትጠቀመው የእፅዋት ክፍል | መጠን |
| --- | --- | --- | --- | --- | --- |
| 1 |  |  |  |  |  |
| 2 |  |  |  |  |  |
| 3 |  |  |  |  |  |
| 4 |  |  |  |  |  |
| 5 |  |  |  |  |  |
| 6 |  |  |  |  |  |
| 7 |  |  |  |  |  |
| 8 |  |  |  |  |  |
| 9 |  |  |  |  |  |
| 10 |  |  |  |  |  |
| 11 |  |  |  |  |  |
| 12 |  |  |  |  |  |

1. ባህላዊ መዳህኒትን በተመለከተ
   1. ባህላዊ መዳህኒትን ማዘጋጃት ከጀምርክ ስንት ዓመት ሆነህ?_________
   2. ይህንን እውቀት ከማን አገኘኸው?_______________________________
2. ለመዳህኒትነት የምትጠቀማቸው እፅዋቶች የትአከባቢ በስፋት ይበቅላሉ?___________ _____________________________________________________________
3. እነዚህ እፅዋቶች በቀላሉ ይገኛሉ? ካልተገኙ ምክንያቱ ምንድ ነው? ________________________________________________________________
4. የመደህኒት አወሳሰዱ እንዴት ነው? (ለተለያዩ በሽታዎች)

____________________________________________________________

1. መዳህኒቱን እንዴት ታዘጋጀዋለህ?_____________________________
2. እፀዋቶቹ ከመዳህኒት ውጪ ለምን ይውላሉ?_____________________________
3. የሰዎች ተፅእኖ በእፅዋት ላይ ምን ይመስላል?________________________
4. በፊት የነበሩ ነገር ግን አሁን የጠፉ እፅዋቶች አሉ? ካሉ ምክንያቱ ምንድ ነው?

_________________________________________________________________

1. ለመዳህኒትነት የሚውሉትን እፅዋቶች በማሳ ወይም በለሌ ቦታ ትተክላቸዋለህ?______
2. ለመዳህኒትነት በሚውሉ እፅዋቶች ላይ ምን አይነት ስጋት አለ ?

________________________________________________________________

1. ለመዳህኒትነት የሚውሉትን እፅዋቶች እንዴት ትንከባክባቸዋለህ ?

________________________________________________________________

1. በአከባቢህ የባህላዊ መዳህኒት እውቀት እንዴት ይተላለፋል?

_______________________________________________________________

ይህንን መረጃ የሰጠሁት መሆኔን በፊረማየ አረጋግጣለሁ

**English version questioners**

The aim of this questioner is to identify the types of animal ailments which occur in the study district, and to identify the effective medicinal plants to treat these ailments. In addition to these, it also important to check the status of conservation habit of the people in the district. Finally, it is important to document medicinal plant knowledge of the people and to plan continuity of this golden knowledge. So please give appropriate answer for these questioners. Thanks in advance for your response.

Self information:

1. Name___________________________ Sex_________ Age_______ Cites_______

Religion: orthodox Muslim Protestant Others

Marital status: Married Single Divorced

Educational status:

Illiterate Informal education Grade 1-8 Grade 9-12

Diploma Degree

1. Questioners regarding health
   1. Is there any animal ailment which occurs in your local environment? ___________. If your answer is yes, please mention each type of aliments and medicinal plants which is used to treat these ailments in the following table.

| No | Types of ailments | Method of transmission | Types of medicinal plants which is important to treat the ailment | Parts of the plant | Dosage |
| --- | --- | --- | --- | --- | --- |
| 1 |  |  |  |  |  |
| 2 |  |  |  |  |  |
| 3 |  |  |  |  |  |
| 4 |  |  |  |  |  |
| 5 |  |  |  |  |  |
| 6 |  |  |  |  |  |

1. Questioners regarding medicinal plants:
   1. When begin preparation of medicinal plants? ______________
   2. From whom you get this knowledge? _______________________
2. From where you harvest these medicinal plants?

___________________________________________________________

1. Is that simple to find this medicinal as you want? If your answer is “no” what is the reason?

____________________________________________________

1. Could you tell me about the application? ______________________
2. How you prepare the remedies?

___________________________________________________________

1. What is the importance of these medicinal plants other than medicine?

____________________________________________________________

1. What you say about the influence human on medicinal plants?

______________________________________________________

1. Is there any plants which extinct currently but present in the previous in this cites? If your answer is “yes” what is the reason for their extinction? ______________________________________________________________
2. Do you have practice of cultivation of the medicinal plant in your farm place? _______________
3. What you say about the threat of medicinal plants in the future?

_____________________________________________________

1. What type action you take to protect medicinal plants?

________________________________________________________________

1. What is method of transmission of medicinal plant knowledge for the next generation in your local environment?

_______________________________________________________________

I assured that this data is given by me. Sign________________
