# Supplementary figures and images for: The study of ethnoveterinary medicinal plants at Mojana Wodera district, central Ethiopia
Source: PLoS One. 2022 May 25;17(5):e0267447. doi: 10.1371/journal.pone.0267447 (PMC9132277; doi:10.1371/journal.pone.0267447)

S2 Appendix: Sample of informant’s response for the questioner


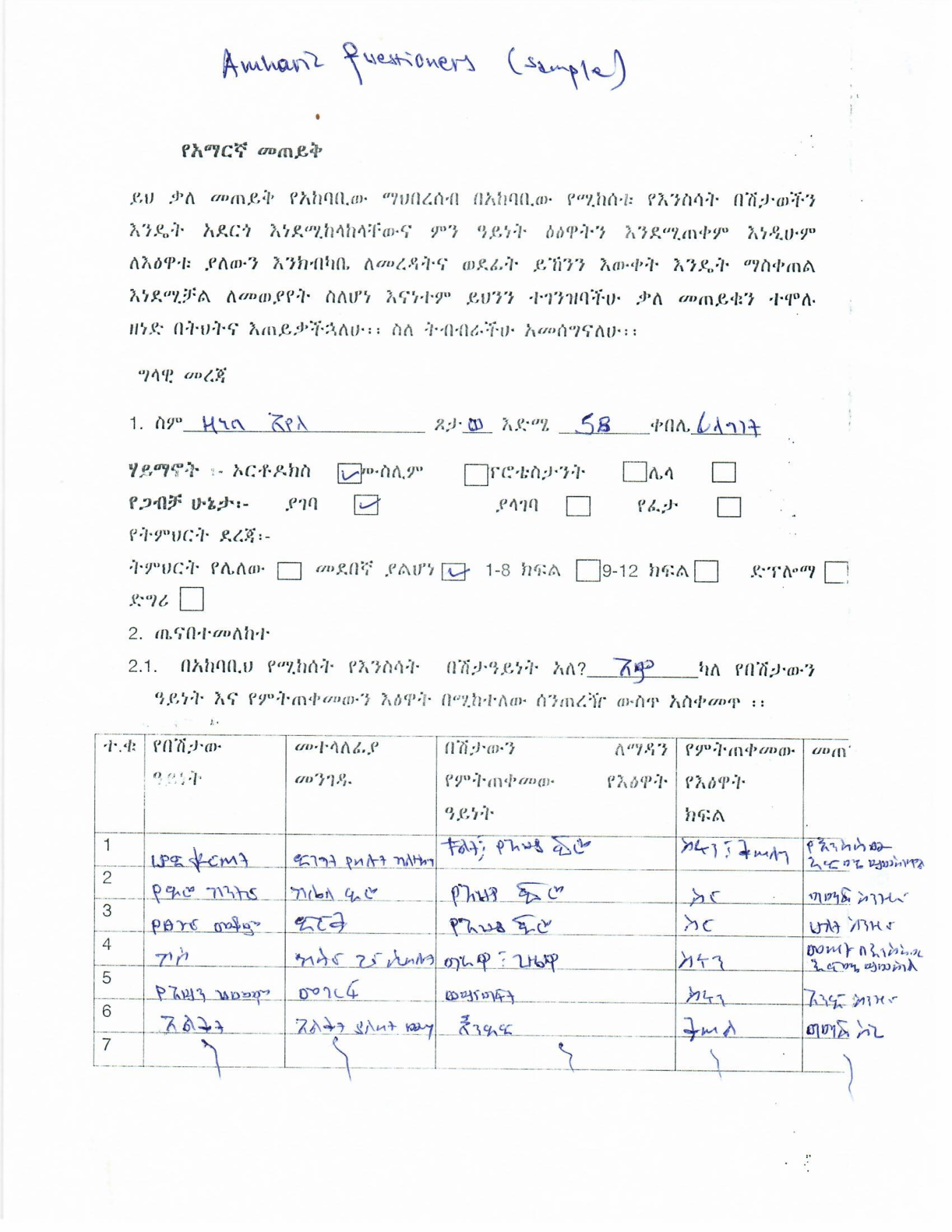

Supplement: S2 Appendix — (DOCX) [file pone.0267447.s004.docx]
